# CONDITIONS ASSOCIATED WITH SUCCESSFUL IMPLEMENTATION OF AN ADVANCE CARE PLANNING INTERVENTION IN NURSING HOMES

**DOI:** 10.1093/geroni/igad104.3244

**Published:** 2023-12-21

**Authors:** Susan Hickman, Edward Miech, Timothy Stump, Laramie Mack, Wanzhu Tu, Kathleen Unroe

**Affiliations:** Indiana University, Indianapolis, Indiana, United States; Indiana University, Indianapolis, Indiana, United States; Indiana University School of Medicine, Indianapolis, Indiana, United States; Regenstrief Institute, Indianapolis, Indiana, United States; Indiana University School of Medicine, Indianapolis, Indiana, United States; Indiana University, Indianapolis, Indiana, United States

## Abstract

Implementing evidence-based interventions in nursing homes is challenging in part because clinical trials requiring a controlled experimental environment are difficult to sustain. In contrast, pragmatic clinical trials develop and evaluate evidence-based interventions in the “real world” with the goal of streamlining implementation after study completion. However, there is minimal information available identifying conditions associated with successful implementation of pragmatic interventions in the nursing home setting. The “Aligning Patient Preferences - a Role Offering Alzheimer’s patients, Caregivers, and Healthcare Providers Education and Support” (APPROACHES) project is a pragmatic trial designed to test and evaluate a staff-led advance care planning (ACP) intervention for residents with ADRD in 131 nursing homes (64 intervention, 67 control) between September 1, 2021 and August 31, 2022. ACP Specialists received training on ACP facilitation and implementation of the ACP program in the facility. Twenty of 65 (31%) sites successfully implemented the APPROACHES intervention and had > 75% of residents with documented ACP conversations. Using configurational analysis, we found two solutions directly linked with successful pragmatic implementation: (1) no executive director turnover during the observation period combined with site participation in monthly intervention support calls with ACP staff at other facilities; and (2) higher rates of hospitalization (3.96 – 7.2 per 1000 resident days) combined with a low number of certified beds. Findings suggest that leadership stability and engagement with peers were essential drivers of successful implementation. Having greater improvement opportunities as well as a more manageable caseload for the ACP Specialist may also help explain successful implementation.

